# Editorial: The role of mushrooms and their nutraceuticals for chronic and degenerative diseases

**DOI:** 10.3389/fphar.2022.1022198

**Published:** 2022-10-11

**Authors:** Sachchida Nand Rai, Mohan P. Singh, Emanuel Vamanu, Haixia Chen

**Affiliations:** ^1^ Centre of Biotechnology, University of Allahabad, Prayagraj, India; ^2^ Faculty of Biotechnology, University of Agricultural Sciences and Veterinary Medicine, Bucuresti, Romania; ^3^ School of Pharmaceutical Science and Technology, Tianjin, China

**Keywords:** mushroom, polysaccharide, *Ganoderma lucidum*, triterpenoids, *Hohenbuehelia serotina*, diabetes mellitus

This Research Topic was on the therapeutic attributes of mushrooms and its bioactive components in various diseases. The first article on this research topic discussed the antidiabetic potential of mushrooms and their polysaccharide components when their oxidative stress is mitigated and the function of β-cells is improved. This review article focused on the role of oxidative stress in the development and progression of diabetes mellitus. The authors suggested that the conventional therapy based on synthetic anti-diabetic drugs shows side effects along with its therapeutic response. They add that polysaccharide-based adjuvant therapy, which has minimal side effects, is commonly used in undeveloped nations. Mushrooms contain a good amount of polysaccharide-based bioactive compounds which have strong anti-oxidative potential. These polysaccharides-based compounds might modulate the activity of β-cells and ultimately improve the diabetic condition in affected individuals. In addition, these mushroom-based polysaccharide adjuvants might work on insulin resistance and interfere with the debilitating processes that hamper the normal physiological activity of affected patients. Several relevant articles from authentic sources like PubMed, Scopus, and Google Scholar, specifically published between 1994 and 2021, are referred to substantiate the main argument in this article. The authors incorporated a table that shows the anti-diabetic properties of 104 different polysaccharides from diverse mushroom species. The antidiabetic activity exhibited by these polysaccharide-based compounds interferes with the activity of reactive oxygen species and reactive nitrogen species. Figure 1 shows the literature search for related information. The authors discussed the pathophysiology and complications of diabetes mellitus and its management with the help of mushrooms and their polysaccharide components by utilizing different signalling pathways. In addition, the authors also discussed the source of different oxidative stress that is connected with diabetic processes. Figure 2 shows the impact of diabetes on multiple organs of the body and Figure 3 sheds light on the role of diabetes and hyperglycemia using different signalling components. The chemical structure of different mushroom-based polysaccharide components is shown in Figure 5. Polysaccharide-based therapeutic response and its mechanism of action, along with signalling pathways behind its anti-diabetic potential, are exhibited in Figure 7. Besides, peroxisome proliferator-activated receptor-γ (PPAR-γ) pathway, polyol pathway, protein kinase c (PKC) pathway, NF-κB PATHWAY, P38 MAPK pathway, and hexosamine pathway are mentioned in this article regarding the anti-diabetic potential of mushroom-based carbohydrate compounds (Arunachalam et al.). The second article on this research topic shows the hypoglycemic effect of natural polysaccharides obtained from *Hohenbuehelia serotina* in type 2 diabetic mouse model. D301/D152 resin ion-exchange chromatography and DEAE-cellulose anion exchange chromatography was used to purify neutral polysaccharides (NHSPs) from mushroom *Hohenbuehelia serotina.* Male BALB/c mice was used in this study to illuminate the antidiabetic potential of NHSPs in the hypoglycemic model. A high sugar/high-fat diet is used in a standard ratio to generate the diabetic mouse model. Fourier Transform Infrared Spectroscopy Analysis and Nuclear Magnetic Resonance Measurements was used in this study. This study revealed that at NHSPs at a dose of 200 mg/kg for 21 days show potent antidiabetic properties by reducing the blood glucose effect (hypoglycemic effect) in type 2 diabetic model (Liu et al.). Nervo-protective, antioxidant, antifungal, antitumor, antiviral, and anti-inflammatory potential of *Ganoderma lucidum* (Gl) was discussed in the third research article by Cör Andrejč et al.. The authors stated that different bioactive components of Gl have exhibited their health-promoting pharmacological and therapeutic effects in various diseases for many years. Sterols, proteins, phenolics, lipids, polysaccharides, and triterpenoids are the most important bioactive components found in Gl and they exhibited strong therapeutic activity with minimal side effects. Antioxidative, anti-inflammatory, anti-bacterial, anti-fungal, and anti-tumor activity of Gl and its bioactive components *in vivo* and *in vitro* studies are discussed in this review article. Methodological quality-related issues, as suggested by the authors, limit the therapeutic activity of GI in cancer. The bioactive activity of triterpenoids and polysaccharides is shown in Figure 1. The structure of β-1, 3-glucan and ganoderic acid is presented in Figure 2. In addition, the common ganoderic acid’s chemical structure from Gl is exhibited in Figure 3. Antitumor activity of triterpenoids on different cell lines was given in Table 1. In other words, the potential therapeutic activity of triterpenoids and carbohydrates from Gl is discussed in this review article (Cör Andrejč et al.). The last article on this research topic advocates the therapeutic application of Gl in the case of emerging diseases in Africa. The authors state the therapeutic properties of GI like anti-inflammatory, anti-microbial, anti-oxidative, anti-histaminic, cytotoxic, and hepatoprotective was well-known; they are time-tested in several countries like Korea, Japan, and China. They further claimed that the Gl and its triterpenoid, carbohydrate-based components and several other bioactive compounds remain unutilized in various parts of Africa. Therefore, there is a strong need to explore the therapeutic potential of Gl and its various bioactive components in various parts of Africa where they are unutilized (Oke et al.).

To conclude, the research articles successfully explore the therapeutic potential of mushrooms, which are considered God’s food, in various diseases like cancer, diabetes, cardiovascular diseases, neurological diseases, etc. The bioactive components present in various mushrooms are responsible for their therapeutic response. Triterpenoids and carbohydrate-based compounds from different mushrooms are the major players involved in the management of different diseases. There is a strong need to explore additional components in diverse verities of mushrooms and explore their therapeutic properties. In addition, future studies will be required to explore mushrooms and their bioactive components specifically in reference to the countries like Africa where mushrooms are not widely used for their therapeutic characteristics.

